# Sexual Expression, Policy, and Practices in Skilled Nursing Facilities: An Updated Assessment in the State of Kansas

**DOI:** 10.1093/geroni/igab046.659

**Published:** 2021-12-17

**Authors:** Sarah Jen, Mijin Jeong

**Affiliations:** 1 University of Kansas, Lawrence, Kansas, United States; 2 University of Kansas, LAWRENCE, Kansas, United States

## Abstract

Prior studies have reported barriers to meeting the sexual needs of older adults within skilled-nursing facilities, such as a lack of privacy, lack of supportive practices and policies, and judgement or discomfort on the part of the staff (Doll, 2013; Hajjar & Kamel, 2003). In 2008, Doll and colleagues assessed the scope of sexual behaviors, staff perceptions of and responses to such behaviors, and whether facilities had a sexual policy in place in SNFs in the state of Kansas (Doll, 2013). In the present study, an online survey was distributed to the same population to provide an updated assessment of sexual behaviors, policies, and practices. Of 60 survey respondents, 62.7% reported knowledge of individual sexual acts (e.g., masturbation) within the past year and 34.5% reported interactional (between two or more residents) sexual acts. When encountering a sexual event, staff were most likely to report the incident to an administrator (76.7%) and treat residents with respect (70.0%), while 35.0% and 41.7% were expected to respond with embarrassment and discomfort, respectively. Only 40% of administrators reported having a policy related to sexual expression. Findings indicate that staff are likely to respond differently to LGBTQ residents due to discomfort and those living with cognitive impairment due to concerns related to consent. The proportion of facilities in Kansas with policies related to sexual expression has increased from 26% to 40% in the past 12 years, but there remains a need for greater specificity of sexuality-related policies and trainings.

